# Evaluation of methods to adjust for treatment switching in clinical trials

**DOI:** 10.1186/1745-6215-12-S1-A139

**Published:** 2011-12-13

**Authors:** Richard Fox, Lucinda Billingham, Keith Abrams

**Affiliations:** 1 Cancer Research UK Clinical Trials Unit, University of Birmingham, Birmingham, B15 2TT, UK; 2 MRC Midland Hub for Trials Methodology Research, University of Birmingham, Birmingham, B15 2TT, UK; 3 Centre for Biostatistics and Genetic Epidemiology, University of Leicester, Leicester LE1 6TP, UK

## Introduction

Treatment switching in clinical trials dilutes estimates of treatment effect which is problematic in decision making, especially in an economic context. In one example 75% of patients crossover from the randomised treatment which emphasises the need to address such bias. We consider methodologies that compensate for this bias.

## Methods

The methods assessed are; intention to treat (ITT), per-protocol (PP), adjusted Cox model [1], causal proportional hazards estimator [2], rank-preserving structural failure-time models (RPSFT) [3], iterative parameter estimation (IPE) [4], parametric randomisation based method [5], and the less well known inverse probability of treatment weighting (IPTW) [6].

## Data

Survival data having an underlying Weibull distribution was simulated and designed such that probability of switching for patient subgroups could be varied within treatment-arms. Other subgroup characteristics could be controlled and 24 scenarios with varied levels of bias were analysed. A review of submissions to the National Institute of Clinical Excellence was performed and used to inform the scenario parameters.

## Results

The RPSFT and IPE methods returned the lowest biases, <8%, in all scenarios. The estimates of the parametric randomisation based method were often far less variable than other methods but were subject to erratic behaviour with extreme biases observed. The other methods performed poorly in general, with biases of up to 50% not uncommon. In particular the IPTW method over-compensates in most scenarios.

## Conclusion

Under these conditions the results clearly identified the RPSFT and IPE methods as most consistent and accurate, with the latter the more consistent of the two. None of the other methods returned consistent results, and as such cannot be recommended.

Further avenues of investigation include exploring the effect of other underlying survival distributions, extending from univariate models to adjust for other covariates, and extending from situations where just control-arm patients switch to scenarios with multidirectional cross-over.
